# Effects of the JC JoyAge Stepped-Care Model Are Differentially Associated With Older Adults’ Baseline Profiles

**DOI:** 10.1093/geroni/igaa057.977

**Published:** 2020-12-16

**Authors:** Tianyin Liu, Gloria Hoi Yan Wong, Shiyu Lu, Terry Lum

**Affiliations:** 1 The University of Hong Kong, Hong Kong, China; 2 hku, Pokfulam, China

## Abstract

Depression is a multidimensional construct consists of different symptom clusters. This paper aims to investigate if the impact of a stepped-care model, JC JoyAge, differs for older adults with different baseline depressive symptom clusters. Data came from 702 older people aged 65 and over who completed the JoyAge program. Their depression (measured by Patient Health Questionnaire-9 [PHQ-9]), anxiety, loneliness, and cognition were assessed by social workers at baseline and 12-months follow-up. Among them 609 were at risk or with mild symptoms and received group-based preventive care (prevention group), and 93 had moderate or above symptoms and received intensive intervention (intervention group). Their responses to PHQ-9 were coded to indicate affective, cognitive, behavioral, and somatic symptom clusters. It was found that somatic complaints had the highest prevalence (91%), followed by affective (83%), behavioral (60%), and cognitive symptoms (41%). Logistic regressions were used to estimate the effects of the program. For the prevention group, the JoyAge preventive care is more effective among those who reported behavioral symptoms (b=0.44, p<0.05, OR=1.55, 95% CI: 1.01, 2.40), but less so in those who had cognitive appraisal issues (b=-0.42, p<0.05, OR=0.66, 95% CI: 0.46, 0.96). For the intervention group, the JoyAge intervention was more effective in treatment among those who reported more affective symptoms (b=0.46, p<0.05, OR=1.59, 95% CI: 1.05, 2.42). The benefits of JC JoyAge stepped-care are differentially associated with participants’ baseline profile. Participants’ overall depressive symptom severity and the presentation of symptom clusters need to be taken into consideration when delivering the services.

